# Fully automatic planning of the long-axis views of the heart

**DOI:** 10.1186/1532-429X-15-S1-O54

**Published:** 2013-01-30

**Authors:** Carmel Hayes, Devos Daniel, Xiaoguang Lu, Marie-Pierre Jolly, Michaela Schmidt

**Affiliations:** 1 Siemens AG, Erlangen, Germany; 2 Ghent University Hospital, Ghent, Belgium; 3 Siemens Corporate Research, Princeton, NJ, USA

## Background

MR imaging of the heart typically involves the acquisition of standard views aligned with the heart axes. Time-efficient and reproducible planning requires expertise in heart geometry and anatomy. In this study, we evaluate the ability of fully automatic heart view localization software to detect the long-axis views of the heart and compare the results both visually and geometrically with manual localization.

## Methods

27 patients (age range 9-70 years) undergoing CMR for a range of different indications (myocarditis, aortic dilatation, ARVD, Turner syndrome) were examined on a 1.5 T MAGNETOM Avanto MR system (Siemens AG, Erlangen, Germany). Following rough localization of the heart within the thorax, a stack of slices covering the entire heart was acquired in the approximate short-axis orientation using a single-shot TrueFISP sequence. This stack of slices was used as input to a machine learning-based algorithm [1] trained with 517 patient datasets which localized and segmented the left ventricle and detected 5 anatomical landmarks. Based on these landmarks, the long-axis views of the heart were calculated automatically and acquired in end-diastole using a single-shot TrueFISP sequence. The user subsequently repeated the long-axis view acquisition following manual landmark positioning. A radiologist, blinded to the acquisition strategy and patient details, retrospectively performed a visual assessment of the automatically and manually planned localizers, grading the overall agreement between the two methods on a scale between 0 and 2 (0 = no difference, 1 = difference but not clinically relevant, 2 = clinically relevant difference) and indicating which views were the best. Angular differences between the two view-planning methods were also calculated.

## Results

**Figure 1:**
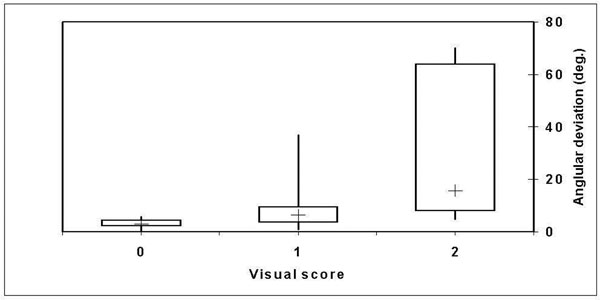
Box-and-whisker plot of the angular deviation (deg.) between the manually and automatically planned views for each of the visual scores (0, 1 and 2).

The automatic view-planning software detected the heart in all 27 cases. Visual agreement scores are summarized in Table 1. In the cases where a clinically relevant difference between the views was observed, i.e. score was 2, automatic view planning was considered better for both the 2- and 4-chamber views (2/27), manual view planning was considered better for the 3-chamber view (3/27). Figure 1 shows box plots of the angular deviation between the automatically and manually planned long-axis views for each of the visual scores. Automatically-planned views were proposed and acquired within 1 minute of the whole heart scout.

**Table 1:** Visual agreement scores for automatic and manual long-axis view planning.

	2-chamber	3-chamber	4-chamber
Score 0	6	1	1
Score 1	19	23	24
Score 2	2	3	2

## Conclusions

We have demonstrated in a group of patients with a range of different cardiovascular pathologies that automatic planning of the long-axis views of the heart can be accomplished in the majority of cases with little or no clinically relevant difference compared with manual planning. Consistent with the visual assessment, the angular differences between the manually and automatically planned views increased with increasing visual difference.

## Funding

Siemens AG.
